# Transmural ischemia visualized using routine Chest CTA^☆^

**DOI:** 10.1016/j.radcr.2022.03.019

**Published:** 2022-03-24

**Authors:** F.Y. van Driest, O Hertgers, A.J.H.A. Scholte, J.W. Jukema, M.A. de Graaf

**Affiliations:** a Department of Cardiology, Leiden Heart-Lung Center, Leiden University Medical Center, Albinusdreef 2, 2333 ZA Leiden, The Netherlands; b Department of Radiology, Leiden University Medical Center, Leiden, The Netherlands

**Keywords:** Computed tomography angiography, CT perfusion, Cardiology, Myocardial infarction, Chest computed tomography

## Abstract

Chest computed tomography (CT) is not routinely used for the diagnosis of myocardial infarction. However, there have been some reports of patients undergoing chest CT for other indications in which ultimately MI was diagnosed due to the presence of areas of myocardial hypoperfusion. The authors present the case of a 60-years old male who is referred due to acute chest pain radiating between the scapulae. Thoracic computed tomography angiography to rule out an aortic dissection revealed an occlusion of the left anterior descending coronary artery with an area of relative hypoperfusion. Hence, the present case demonstrates how using routine thoracic computed tomography angiography, transmural ischemia can be visualized in the setting of an acute myocardial infarction. It should make clinicians aware of the fact it may be beneficial to look for myocardial perfusion abnormalities when assessing chest CT's.

## Introduction

Myocardial infarction (MI) is usually diagnosed by the presence of specific clinical symptoms such as chest pain, new ischemic electrocardiogram (ECG) changes, elevated cardiac biomarkers and potentially new regional wall motion abnormalities as diagnosed by echocardiography [1]. Chest computed tomography (CT) is not regarded as a necessary imaging modality in the routine evaluation of MI. However, there have been reports of patients in which MI has been detected on chest CT as areas of myocardial hypoperfusion. Such findings are not uncommon in patients being evaluated for other indications such as aortic dissection and pulmonary embolisms [2]. CT scanning allows for a fast and non-invasive imaging modality. Also, with the ever-increasing improvement of spatial and temporal resolutions chest CT may be very beneficial in evaluating patients with acute MI [3].

## Case report

A 60-years old man was admitted to our emergency department due to acute chest pain radiating between the scapulae. Patient had no prior medical history. Physical examination revealed no abnormalities aside from hypertension, with a significant inter-arm difference (140/90mmhg left & 130/80mmhg right). Electrocardiogram (ECG) demonstrated a normal sinus rhythm and ST-elevations in V1-V2 with no other abnormalities. Because of the pain between the scapulae and difference in blood pressure, first a thoracic computed tomography angiography (CTA) was performed using a double rule-out protocol tailored to rule out aortic dissection and pulmonary embolism. The scan was performed using a 320-row volumetric scanner (Aquilion ONE, Canon Medical Systems, Otawara, Japan). All images were analyzed using dedicated post-processing software (Vitrea FX 7.12; Vital Images, Minnetonka, Minnesota). There were no visual signs of aortic dissection. However, an occlusion of the left anterior descending artery (LAD) in the middle segment was observed with limited contrast opacification of the distal segment (Panel A and B). Visual inspection of the myocardium using dedicated window-settings revealed an area of relative hypoperfusion of the apical and anterior segments, consistent with transmural ischemia in the LAD-territory. (panel C and D). Further visualization using the transmural perfusion ratio reveals a large area of relative hypoperfusion extending over the anterior wall reaching the apex (panel E). This was confirmed using echocardiography (supp material) demonstrating moderate Left-ventricular function (LVEF 36%) with an akinetic aneurysm of the apex and akinesia in the basal and mid anteroseptal region. Invasive angiography revealed a total occlusion of the left anterior descending artery middle segment (panel F) for which successful percutaneous coronary intervention (PCI) was performed.

**Fig. 1:**
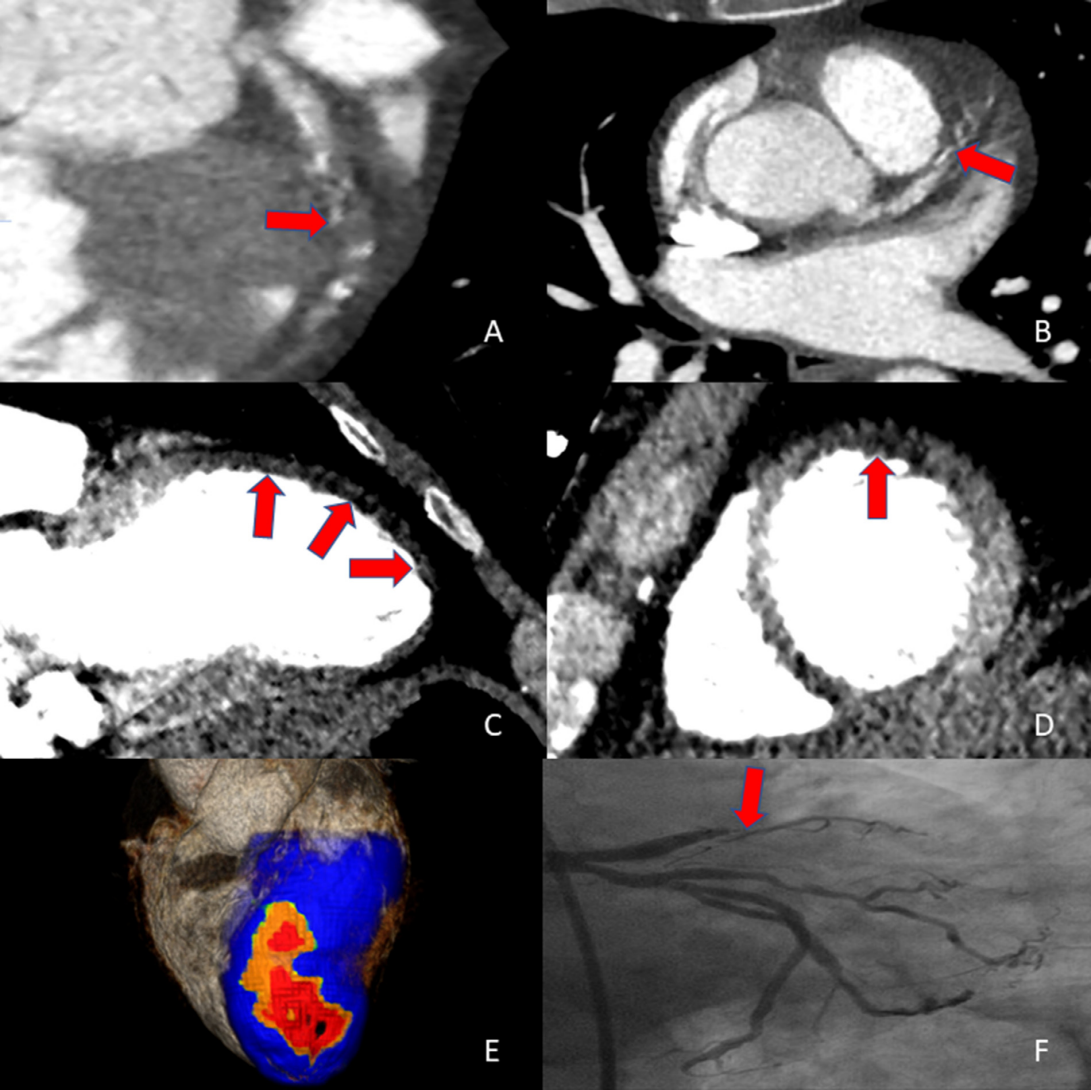
An occlusion of the left anterior descending artery (LAD) in the middle segment is observed with limited contrast opacification of the distal segment (Panel A and B). Visual inspection of the myocardium using dedicated window-settings reveals an area of relative hypoperfusion of the apical and anterior segments, consistent with transmural ischemia in the LAD-territory. (panel C and D). Further visualization using the transmural perfusion ratio reveals a large area of relative hypoperfusion extending over the anterior wall reaching the apex (panel E). Invasive angiography revealed a total occlusion of the left anterior descending artery middle segment (panel F)

## Discussion

Dual rule-out Chest CT is not commonly used for the evaluation of acute MI. However, it has been proven to be very useful in diagnosing aortic dissection and pulmonary embolisms [4]. A major downside of the aforementioned protocol in the visualization of MI is that visualization of perfusion abnormalities also requires appropriate timing of scanning relative to the administration of intravenous contrast. For instance, the protocol regarding pulmonary arteries timing may be too early to show perfusion deficits [5].

Advancements in CT technology have enhanced the visualization of MI on routine chest CT's especially by decreasing the prevalence of beam hardening artifacts [6]. In a study by Gosalia et al. 18 patients presenting with chest discomfort and primary scan indications of pulmonary malignancy, pulmonary embolism, or aortic dissection, MI was incidentally found in 15 patients yielding a sensitivity and specificity of 83% and 95% respectively. Hence, it was demonstrated that an initial acute MI is detectable on routine contrast-enhanced chest CT as areas of myocardial hypoperfusion [2]. Although detection of MI using chest CT seems promising it must be emphasized that conventional cardiac CT remains the number one modality of choice as the visualization of myocardium is superior [2].

The present case demonstrates how, a non-cardiac CTA revealed acute myocardial infarction by visualizing a perfusion deficit in the vascular territory of the left coronary artery. This unexpected finding probably suggests a new role for routine chest CTA as transmural ischemia can be visualized in the setting of an acute myocardial infarction. Close inspection of the myocardium with dedicated window-settings, can aid in the diagnostic work-up of patients with acute chest pain.

## Patient consent

We hereby confirm that written, informed consent for publication of their case was obtained from the patient.

## Appendix Supplementary materials

Image, video 1
